# Uremic serum residue decreases SN-38 sensitivity through suppression of organic anion transporter polypeptide 2B1 in LS-180 colon cancer cells

**DOI:** 10.1038/s41598-019-51640-9

**Published:** 2019-10-29

**Authors:** Shoichi Ozawa, Masayuki Tsujimoto, Hitoshi Uchiyama, Natsuko Ito, Satoe Morishita, Mizuki Yamamoto, Ryosuke Irie, Tohko Sakashita, Hidehisa Tachiki, Taku Furukubo, Satoshi Izumi, Tomoyuki Yamakawa, Tetsuya Minegaki, Kohshi Nishiguchi

**Affiliations:** 1 Department of Clinical Pharmacy, Faculty of Pharmaceutical Science, Kyoto Pharmaceutical University, 5 Misasagi Nakauchi-cho, Yamashina-ku, Kyoto 607-8414 Japan; 2 Scientific Research and Business Development Department, Towa Pharmaceutical Co. Ltd., Kyoto Research Park KISTIC#202, 134 Chudoji Minami-Machi, Shimogyo-ku, Kyoto 600-8813 Japan; 3 Department of Pharmacy Service, Shirasagi Hospital, 7-11-23 Kumata, Higashisumiyoshi-ku, Osaka 546-0002 Japan; 4 Department of Medicine, Shirasagi Hospital, 7-11-23 Kumata, Higashisumiyoshi-ku, Osaka 546-0002 Japan

**Keywords:** Kidney diseases, Cancer therapeutic resistance

## Abstract

Pharmacokinetics of SN-38 in patients with end-stage kidney disease (ESKD) is partially varied because of fluctuations in transporters expression and/or function by high protein bound-uremic toxins concentration. The fluctuations may induce variations in anticancer drugs sensitivity to cancer cells. We aimed to clarify the variations in sensitivity of SN-38 to cancer patients with ESKD and investigate this mechanism, by human colon cancer cells exposed to uremic serum residue. LS180 cells were exposed to normal or uremic serum residue (LS/NSR or LS/USR cells) for a month. IC_50_ values of SN-38 in LS/NSR or LS/USR cells were calculated from viability of each cells treated SN-38. mRNA expression and intracellular SN-38 accumulation was evaluated by RT-PCR and HPLC-fluorescence methods, respectively. The IC_50_ value in LS/USR cells was higher than that in LS/NSR cells. Organic anion transporter polypeptide (OATP) 2B1 mRNA expression was lower in LS/USR cells than in LS/NSR cells, and SN-38 accumulation in LS/USR cells was lower than that in LS/NSR cells. Only co-treatment baicalin, which is OATP2B1 inhibitor, almost negated the difference in SN-38 accumulation between LS/NSR and LS/USR. Anticancer effects of substrates of OATP2B1, such as SN-38, were reduced in ESKD patients at the same plasma substrate concentration.

## Introduction

Irinotecan tend to be prescribed to colorectal cancer patients with renal failure because irinotecan is known as type of metabolite medicine by liver. In addition, its active metabolite (SN-38) is metabolized via glucuronidation by UDP-glucuronosyltransferase in hepatocytes. In other words, from viewpoint of the pharmacokinetic, irinotecan is theoretically more applicable for renal dysfunction patients than oxaliplatin, which is type of renal excretion and also to administrate colorectal cancer patients, when to consider chemotherapy. However, kidney dysfunction is reported to be a risk factor of neutropenia and thrombocytopenia in patients undergoing irinotecan-based chemotherapy^1^. In addition, it has been also reported that the clearance of SN-38 after the administration of irinotecan is remarkably delayed in patients with end-stage kidney disease (ESKD), the last stage of chronic kidney disease, even though SN-38 is mainly excreted by the liver^2^. Previously, we have reported that serum residue in patients with ESKD inhibits the uptake of SN-38 through organic anion transporter polypeptide (OATP) 1B1^3^. Therefore, the serious hematotoxicity and extended half-life of SN-38 may be caused, at least in part, by the dysfunction of OATPs in hepatocytes.

Protein bound-uremic toxins accumulate in patients with ESKD^4^. These uremic toxins have been reported to affect the expression and function of drug transporters. For example, Reyes *et al*.^5^ indicated that quinoline acid and indole-3-acetic acid inhibited the uptake of estron-3-sulfate through OATP1B1 and OATP1B3 and the uptake of losartan into rat hepatic cells. They also indicated that the serum of patients with ESKD inhibited the uptake of losartan into human hepatic cells^5^. In addition, we reported that the simultaneous treatment of serum and digoxin in patients with ESKD inhibits the uptake of digoxin into isolated human hepatocytes^6^, and that pretreatment of human hepatocellular carcinoma Hep3B cells with serum from patients with ESKD decreases the initial uptake of pravastatin and the expression of OATP2B1 mRNA^7^. These reports indicate that the variation in hepatic clearance was partially induced by fluctuations in transporter expression and/or function in patients with ESKD.

The functional variation in transporters also induces the resistance of cancer cells to anticancer drugs. For example, it has been reported that high expression of efflux transporters, such as multidrug resistance protein (MDR) 1 and breast cancer resistance protein (BCRP), induces resistance to several chemotherapy^8^. In addition, Yang L *et al*.^9^ showed that indoxyl sulfate enhanced the expression of BCRP, one of the determinant factors of the anticancer effects of SN-38, in Caco-2 cells. In contrast, it was reported that the high expression of uptake transporter OATP1B3 improved the 5-year survival rate for patients with colorectal cancer^10^. Therefore, there is a concern that the functional variation of transporters in patients with ESKD affects not only pharmacokinetics, but also sensitivity to anticancer drugs. Some reports about OATP2B1 and SN-38 have already been reported. Fujita *et al*. have shown that SN-38 is a substrate of OATP2B1^11^. We have previously demonstrated that uremic serum residue down-regulates OATP2B1 expression on Hep3B^7^ and uremic serum residue decline functions of OATP1B1 when to carry SN-38 into HEK293 expressing OATP1B1^3^. However, these reports do not demonstrate directly that uremic serum affect SN-38 sensitivity in colon cancer. Our hypothesis is that these fluctuations of transporter function also occur in cancer cells, and that the anticancer effects of SN-38 can vary in patients with ESKD. Nevertheless, the evaluation of uremic serum, including the effect of uremic toxins on SN-38 sensitivity, has not been conducted.

The aim of this study was to clarify the effects of variations in SN-38 sensitivity in patients with ESKD and cancer; therefore, we evaluated the variations in SN-38 sensitivity and the mechanisms in human colon cancer cells exposed to uremic serum residue.

## Results

### Difference in sensitivity of LS/NSR and LS/USR cells to SN-38

**Figure 1:**
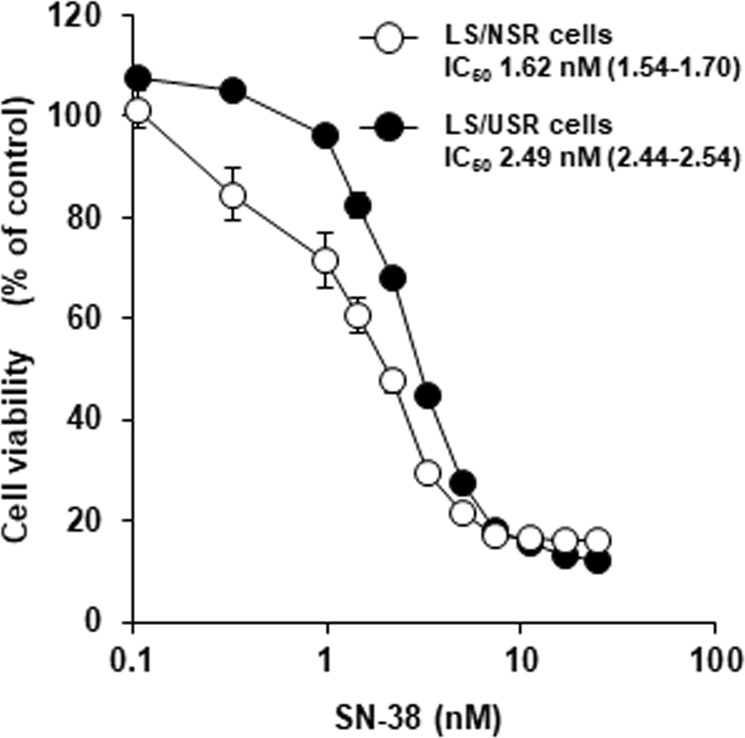
Difference in sensitivity of LS180 cells were exposed to normal serum residue (LS/NSR) and uremic serum residue (LS/USR) to SN-38. LS/NSR (○) and LS/USR cells (●) were seeded into 96-well plates (1.0 × 10^3^ cells/well in 100 µL of medium) and cultured for 24 h, and then were exposed to SN-38 (0–25 nM) for 6 days. The cell viability was assessed using the Quanti Blue^TM^ Cell Viability Assay Kit. Each point represents the mean ± S.E. (n = 4). IC_50_ values represent the estimated value (95% confidence interval).

The survival curve for LS/USR cells was shifted to the high SN-38 concentration side compared with that for LS/NSR cells. The IC_50_ value of SN-38 in LS/USR cells (2.49 nM, 95% CI: 2.44–2.54 nM) was significantly higher than that in LS/NSR cells (1.62 nM, 95% CI: 1.54–1.70 nM) (Fig. 1).

### Difference in SN-38 uptake between LS/NSR and LS/USR cells

**Figure 2:**
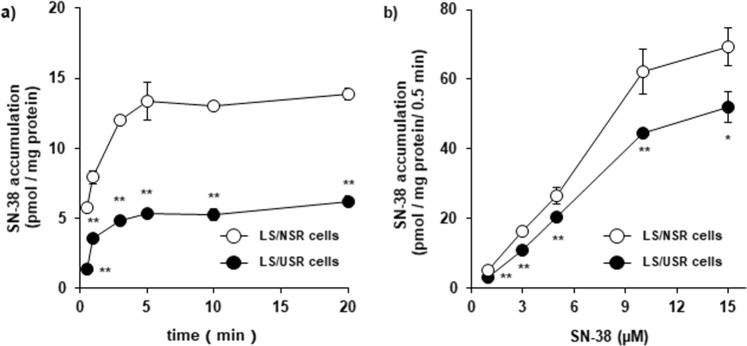
Difference in SN-38 accumulation between LS180 cells were exposed to normal serum residue (LS/NSR) and uremic serum residue (LS/USR). LS/NSR (○) and LS/USR cells (●) were seeded at 1.2–1.5 × 10^5^ cells/well into 24-well plates. (**a**) The accumulation of SN-38 was determined after incubation with SN-38 (1 µM) at 37 °C for 0.5, 1, 3, 5, 10, and 20 min. (**b**) The accumulation of SN-38 was determined following incubation with SN-38 (1, 3, 5, 10, and 15 µM) at 37 °C for 0.5 min. Each point represents the mean ± S.E. (n = 3–4). The significance of any differences between the mean values was determined by unpaired Student’s *t*-test (**p < 0.01, *p < 0.05).

The uptake of SN-38 was increased over time up to 5 min (Fig. 2a) and was dependent on SN-38 concentration up to 10 µM in both cells (Fig. 2b). In addition, these uptakes were saturated at high SN-38 concentration (Fig. 2b), and the uptake of SN-38 into LS/USR cells was significantly lower than that in LS/NSR cells under any conditions (Fig. 2).

### Difference in expression of drug transporters between LS/NSR and LS/USR cells

**Figure 3:**
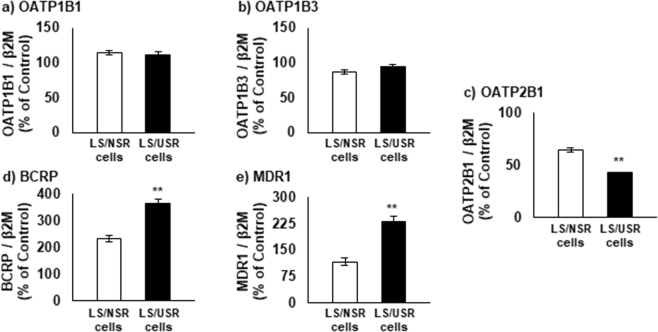
Difference in mRNA expression of drug transporters between LS180 cells were exposed to normal serum residue (LS/NSR) and uremic serum residue (LS/USR) and LS180 cells were exposed to uremic serum residue (LS/USR cells). LS/NSR (□) and LS/USR cells (■) were seeded at 3.0 × 10^5^ cells/well into 6-well plates. The expression of organic anion transporting polypeptide (OATP)1B1 (**a**), OATP1B3 (**b**), OATP2B1 (**c**), breast cancer resistance protein (BCRP) (**d**), and multidrug resistance protein (MDR) 1 mRNA (**e**) was quantified by real-time RT-PCR. Each column represents the mean ± S.E. (n = 3–4). The significance of any differences between the mean values were determined by unpaired Student’s *t*-test (*p < 0.05, **p < 0.01).

There was no difference in OATP1B1 and OATP1B3 mRNA between LS/NSR and LS/USR cells (Fig. 3a,b). In contrast, BCRP and MDR1 mRNAs in LS/USR cells were expressed more highly than in LS/NSR cells (Fig. 3d,e), and OATP2B1 mRNA in LS/USR cells was less strongly expressed in LS/NSR cells (Fig. 3c).

### Effects of Ko143 and/or verapamil on the uptake of SN-38 into LS/NSR and LS/USR cells

**Figure 4:**
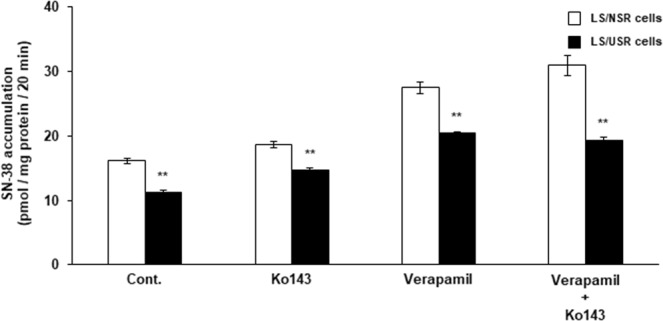
Effects of Ko143 and/or verapamil on the accumulation of SN-38 into LS180 cells were exposed to normal serum residue (LS/NSR) and uremic serum residue (LS/USR). LS/NSR (□) and LS/USR cells (■) were seeded at 1.2–1.5 × 10^5^ cells/well into a 24-well plate. The accumulation of SN-38 was determined following incubation with SN-38 (1.0 µM) in the absence (Cont.) or presence of Ko143 (500 nM) and/or verapamil (200 µM) at 37 °C for 20 min. Each column represents the mean ± S.E. (n = 4–6). The significance of differences between the mean values was determined by unpaired Student’s *t*-test (**p < 0.01).

Co-treatment with verapamil and/or Ko143 increased the uptake of SN-38 into both cell types, but did not affect the difference in SN-38 uptake between LS/NSR and LS/USR cells (Fig. 4).

### Effects of rifampicin and/or baicalin on the uptake of SN-38 into LS/NSR and LS/USR cells

**Figure 5:**
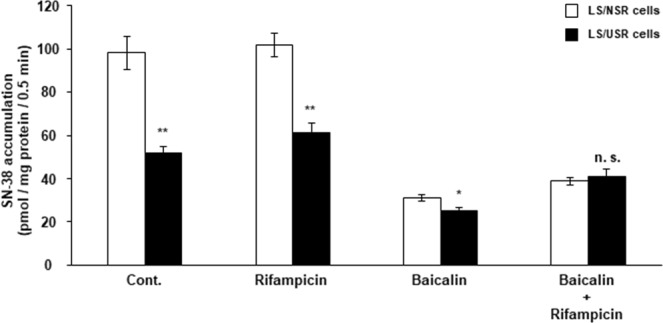
Effects of rifampicin and/or baicalin on the accumulation of SN-38 into LS180 cells were exposed to normal serum residue (LS/NSR) and uremic serum residue (LS/USR). LS/NSR (□) and LS/USR cells (■) were seeded at 1.2–1.5 × 10^5^ cells/well into 24-well plates. The accumulation of SN-38 was determined following incubation with SN-38 (10 µM) in the absence (Cont.) or presence of rifampicin (100 µM) and/or baicalin (1 mM) at 37 °C for 0.5 min. Each column represents the mean ± S.E. (n = 3–4). The significance of differences between the mean values was determined by an unpaired Student’s *t*-test (*p < 0.05, **p < 0.01, N.S. = no significance).

Rifampicin did not affect the uptake of SN-38 into LS/NSR and LS/USR cells. In contrast, baicalin clearly decreased the uptake of SN-38 into both cells, and almost negated the difference in the uptake of both cell types (Fig. 5).

## Discussion

In this study, it has been shown that the long-term exposure to USR significantly reduced the sensitivity to SN-38 in LS180 cells (Fig. 1), and the mechanism may be the decreased uptake on SN-38 owing to downregulation of the uptake transporter (Figs 2 and 5). These results suggested that the sensitivity of cancer cells to SN-38 may be decreased in patients with ESKD. Therefore, it is important to be concerned about the decline of renal and nonrenal clearance, but also the decrease in sensitivity to anticancer drugs; therefore, there is a need for careful monitoring of the antitumor effects in patients with ESKD.

The expression of not only OATP1B1 and 1B3, but OATP2B1 mRNA in LS/USR cells was lower than in LS/NSR cells (Fig. 3). In addition, not rifampicin, an OATP1B1 and OATP1B3 inhibitor, but baicalin, an OATP2B1 inhibitor, almost negated the difference in SN-38 uptake between LS/NSR and LS/USR cells (Fig. 5). As SN-38 is a known substrate of OATP2B1^11^, the decline in sensitivity to SN-38 in LS/USR cells is caused by the downregulation of OATP2B1 expression. These results suggested that the sensitivity to anticancer drugs, such as substrates of OATP2B1 like SN-38, may be decreased in patients with ESKD. This is the first study to show the possibility of decreased sensitivity to anticancer drugs in patients with ESKD.

In this study, it was shown that USR decreased OATP2B1 mRNA expression (Fig. 3c). It has been reported that OATP2B1 expression is partially regulated by thyroid hormone receptor (TR), one of the transcription factors. Henriette *et al*.^12^ reported that triiodothyronine and thyroxine induced the expression of OATP2B1 mRNA. The promoter region of OATP2B1 has direct repeats spaced by four nucleotides (DR-4) and a TR bound sequence. In addition, Santos *et al*.^13^ reported that serum before dialysis significantly decreased luciferase activity on the cDNA-inserted DR-4 domain, compared with serum after dialysis, in patients after ESKD. Therefore, our results suggested that serum compounds, which can be eliminated by dialysis, downregulated the expression of OATP2B1 mRNA.

Although it was shown that MDR1 mRNA expression was higher in LS/USR cells than in LS/NSR cells, although the MDR1 inhibitor verapamil did not affect the difference in SN-38 uptake of both cells (Fig. 4). Jansen *et al*.^14^ have reported that the overexpression of MDR1 did not affect SN-38 sensitivity. Therefore, it cannot be denied that MDR1 is induced in patients with ESKD, although the induction of MDR1 mRNA expression did not affect the decreased sensitivity to SN-38 in patients with ESKD.

Ko143, a BCRP inhibitor, did not affect the difference in SN-38 uptake in LS/NSR cells and LS/USR cells, even though BCRP mRNA in LS/USR cells was significantly higher than that in LS/NSR cells. These results indicated that the induction of BCRP did not contribute to the decrease in SN-38 sensitivity caused by USR. In general, the overexpression of BCRP decreased SN-38 sensitivity^9^. Mutsaers *et al*.^15^ reported that indoxyl sulfate and hippuric acid noncompetitively inhibited BCRP-mediated estron-3-sulfate uptake into inside-out vesicles from cells overexpressing BCRP, and K_i_ and IC_50_ of indoxyl sulfate are 500 µM and 770 ± 120 µM and K_i_ and IC_50_ of hippuric acid are 3670 ± 170 µM and 4000 ± 100 µM, respectively. We have previously shown uremic toxins concentration, indoxyl sulfate (total: 163.8 ± 6.2 µM, unbound: 26.3 ± 2.4 µM) and hippuric acid (total: 208.7 ± 2.6 µM, unbound 106.1 ± 1.5 µM) (Values represent the mean ± S.E.)^16^. These K_i_ and IC_50_ are higher than concentration of uremic toxins in our uremic serum, in other words, BCRP seems not to be inhibited by sole uremic toxin, but uremic toxins and other components in uremic serum may co-inhibit that. Therefore, future studies should be conducted to consider the interaction between SN-38, uremic toxins and other components, and the induction and inhibition of BCRP.

This study has showed that USR decreased the expression of OATP2B1 mRNA (Fig. 3cs) and the sensitivity to SN-38, the substrate of OATP2B1 (Fig. 1). Because some anticancer drugs, such as erlotinib, are a substrate of OATP2B1^17^, the sensitivity to these anticancer drugs may be decreased in patients with ESKD. However, OATP2B1 is reported to be expressed in both cancer cells and hepatocytes^18^; in addition, we have reported that USR decreased the expression of OATP2B1 mRNA in Hep3B cells^7^. The plasma concentration of these anticancer drugs may increase as the hepatic uptake is decreased by the downregulation of OATP2B1 expression in hepatocytes, and the increase most likely offsets the decreased susceptibility of patients with ESKD.

This study has some limitations. First, as we did not conduct *in vivo* research, there is a need for further research using animal models with ESKD and cancer. The *in vivo* research should provide more information such as the difference of sensitivity in relation to the calculated renal clearance or to concentration of uremic toxin, hormone or waste in blood. Second, this study did not research other factors, such as UDP-glucuronosyltransferase (UGT) 1A1. UGT1A1 is expressed in colorectal cancer, and p-cresol and indoxyl sulfate inhibit the function of UGT1A1^19,20^; therefore, further research is needed to evaluate the effects of USR on the function of UGT1A1. Third, we demonstrated different function of OATP2B1 in LS/NSR and LS/USR in uptake study, however we did not confirm protein level in each cells. It is necessary to discover the mechanism in molecular biology level in future study. Lastly, we used only LS180 to demonstrate SN-38 sensitivity in colorectal cancer patients with ESKD. Because of restriction to use uremic serum in our study due to ethical concern and the amount, we could do our research by only one cell line, LS180. Even though LS180 is a good model for our study since some clinical samples are reportedly recognized OATP2B1 expression on themselves^21^, it is needed to demonstrate by other colorectal cancer cell line or tissue to confirm reproducibility in future study.

## Conclusion

In conclusion, long-term exposure to USR decreased the sensitivity of LS180 cells to SN-38 through the downregulation of OATP2B1 mRNA and a reduction in the uptake of SN-38. These results suggested that the anticancer effects of substrates of OATP2B1, such as SN-38, decreased in patients with ESKD at the same plasma concentration. Therefore, not only the increase in SN-38 concentration, but also the decrease in the antitumor effects of SN-38 in patients with ESKD, should be considered.

## Methods

### Materials

Camptothecin and SN-38 were purchased from Tokyo Chemical Industry Co., Ltd. (Tokyo, Japan). Ko143 was purchased from Sigma Aldrich Co. LLC (St. Louis, MO, USA). Verapamil and rifampicin were purchased from FUJIFILM Wako Pure Chemical Corporation (Osaka, Japan). Baicalin was purchased from AdooQ Bioscience (Irvine, CA, USA), and Cell Quanti-Blue^TM^ Cell Viability Assay Kit was purchased from Bio Assay Systems (Hayward, CA, USA). All other reagents were of high-purity analytical or high-performance liquid chromatography (HPLC)-grade. Pooled human serum from healthy subjects (normal serum) was purchased from Merck Millipore Ltd. (Billerica, MA, USA), and pooled human serum from patients with ESKD (uremic serum) was obtained from more than 400 patients with ESKD undergoing hemodialysis at Shirasagi Hospital (Osaka, Japan). Blood samples were collected immediately before hemodialysis in a non-invasive manner. Normal and uremic serum were deproteinized by 3-times volume of methanol as much as serum and evaporated under a nitrogen stream at 50 °C; the resulting serum residues, normal serum residue (NSR) and uremic serum residue (USR), were used in the study^3^. The present study was approved by the ethics committees of Shirasagi Hospital (IRB number J2012013) and Kyoto Pharmaceutical University (IRB number 08-04).

### Cell culture

A stock of the intestinal human colon adenocarcinoma LS180 cell line was purchased from American Type Culture Collection (Manassas, VA, USA). LS180 cells were cultured in Dulbecco’s modified Eagle’s medium (DMEM, Invitrogen Co. Grand Island, NY, USA) supplemented with 10% fetal bovine serum (Lot. AWD12933 and AXM56561, Thermo Fischer Scientific, Waltham, MC, USA), 100 U/mL penicillin and 100 μg/mL streptomycin (Invitrogen Co. and Nacalai Tesque, Inc., Kyoto, Japan), and 0.1 mM non-essential amino acids (Invitrogen Co. and Nacalai Tesque, Inc.). NSR and USR were dissolved in DMEM as much as 10 times volume of serum and passed through a 0.22 µm membrane filter to sterilize. LS180 cells were cultured for 1 month in DMEM with NSR (NSR-DMEM) or USR (USR-DMEM); these cells were as named LS/NSR cells and LS/USR cells, respectively. For evaluation of sensitivity to SN-38 in LS180 cells, LS/NSR cells and LS/USR cells were seeded into 96-well plates (1 × 10^3^ cells/well) for a day. For uptake and efflux studies, both of cells were seeded into 24-well plates (1.2–1.5 × 10^5^ cells/well) for 3–4 days. For real-time RT-PCR, both of cell were seeded into 6-well plates (3 × 10^5^ cells/well) for 3–4 days. All of culturing conditions were in a humidified atmosphere containing 5% CO_2_ at 37 °C.

### Evaluation of sensitivity to SN-38 in LS180 cells

After removal of DMEM, each well was exposed to SN-38 (0.1–25 µM) dissolved in NSR-DMEM or USR-DMEM for 6 days. After 6 days, the cell viability was measured by Cell Quanti-Blue^TM^ at the excitation and emission wavelengths of 535 nm and 590 nm, respectively, by using a microplate reader (Power Scan^®^ HT, DS Pharma Biomedical Co., Ltd., Osaka, Japan). The 50% inhibitory concentration (IC_50_) was calculated by using the non-linear least squares program (MULTI) from the equation I = I_max_ × (1 − C^γ^/(C^γ^ + IC_50_^γ^)), where I, I_max_, C, and γ are cell viability (percentage of control), maximum cell lethality, drug concentration in the medium, and sigmoid function, respectively.

### Uptake buffer

SN-38 was dissolved in a 1:1 mixture of 50 mM NaOH and dimethyl sulfoxide (DMSO) for 24 h before the uptake experiments in order to convert SN-38 to its carboxylate form^22^. SN-38 dissolving DMSO was mixed in Hanks Balanced Salt Solution (HEPES-HBSS: 136.9 mM NaCl, 5.4 mM KCl, 1.3 mM CaCl_2_, 0.4 mM MgSO_4_, 0.3 mM Na_2_HPO_4_, 0.4 mM KH_2_PO_4_, 4.2 mM NaHCO_3_, 5.56 mM glucose, and 25 mM HEPES) at 1.0 µM SN-38 with 0.5% DMSO, for a time-dependency and an efflux transporter studies, at 1.0, 3.0, 5.0, 10, and 15 µM SN-38 with 0.5% DMSO for a concentration-dependency study, and at 10 µM SN-38 with 0.5% DMSO, for a influx transporter study. In the efflux transporter study, 500 nM Ko143, a specific BCRP inhibitor^23^, and/or 200 µM verapamil, a specific MDR1 inhibitor^24^, were dissolved in HEPES-HBSS containing SN-38. In the influx transporter study, 100 µM rifampicin, a specific inhibitor of OATP1B1 and OATP1B3 inhibitor^25^, and 1 mM baicalin, a specific OATP2B1 inhibitor^11^, were dissolved in HEPES-HBSS containing SN-38.

### Uptake study

The cells were washed twice with 37 °C fresh HEPES–HBSS, and preincubated with HEPES-HBSS 500 µL for 10 min at 37 °C. The uptake experiments were initiated by the replacement of HEPES-HBSS with 300 µL HEPES–HBSS dissolved SN-38. After 0.5, 1, 3, 5, 10, and 20 min (time-dependency experiment), or 0.5 min (concentration-dependency and influx transporter experiments), the uptake experiments were terminated by the aspiration of the SN-38 solution. Subsequently, the cells were washed twice with phosphate-buffered saline (137 mM NaCl, 2.7 mM KCl, 1.5 mM KH_2_PO_4_, and 8.1 mM Na_2_HPO_4_) containing 0.5 mM MgCl_2_ and 1 mM CaCl_2_ (PBS (+)), and three times with ice-cold PBS (+). After the experiment, the cells were stored at −30 °C until the SN-38 content was quantified.

### Efflux study

The cells were washed twice with 37 °C fresh HEPES–HBSS and preincubated with 500 µL HEPES-HBSS for 10 min at 37 °C. Subsequently, the cells were incubated in 500 µL HEPES-HBSS dissolved Ko143 and/or verapamil. After 20 min, the efflux experiments were initiated by the replacement of the preincubation solution with 300 µL HEPES–HBSS containing SN-38 and Ko143 and/or verapamil. After 20 min, the efflux experiments were terminated by the aspiration of HEPES-HBSS. Subsequently, the cells were washed twice with PBS (+) and three times with ice-cold PBS (+). After the experiment, the cells were stored at −30 °C until the SN-38 content was quantified.

### Quantification of SN-38

SN-38 was quantified by using HPLC with a fluorescence detector (RF-20Axs; Shimadzu Corporation, Kyoto, Japan; excitation, 370 nm; emission, 530 nm). The frozen cells were dissolved in 0.4 M NaOH and neutralized with 0.4 M HCl. Acetonitrile (400 or 1200 μL), 5 ng/mL camptothecin as an internal standard in methanol (60 µL), and 0.02 M HCl (30 µL or 120 µL) to convert SN-38 to lactone^26^ were added to the samples (100 or 300 µL). The mixture was centrifuged at 10,000 *g* for 3 min and evaporated to dryness under a stream of N_2_ at 40 °C. The residue was dissolved in 200 µL of the mobile phase (a 75:25 (v/v) ratio of 20 mM citrate buffer, pH 3.0: acetonitrile) and analyzed by using HPLC (LC-20AT; Shimadzu Corporation, Kyoto, Japan) on an Inertsil ODS III column (4.0 mm I.D. × 250 mm × 4 mm, 5 μm; column temperature, 40 °C; GL Sciences Inc., Tokyo, Japan) at a flow rate of 1.0 mL/min. Each result was standardized to the protein content in each sample. Protein content was determined by using Lowry’s method^27^ with bovine serum albumin as the standard.

### Real-time RT-PCR

Total RNA was extracted from the cells by RNAzol^®^ RT Reagent (Molecular Research Center, Inc., Cincinnati, OH, USA). The absorbance was measured by using DU^®^ 730 (Beckman Coulter, Tokyo, Japan), and the concentration of RNA was calculated from the equation Total RNA (ng/mL) = A_260_ × 40. cDNA was synthesized from 1 µg of total RNA by a reverse transcriptase (RT) reaction using ReverTra Ace^®^ qPCR (Toyobo Co., Ltd., Osaka, Japan) and i-Cycler iQ (Bio-Rad Laboratories, Inc., Hercules, CA, USA). Total RNA was heated at 37 °C for 15 min (RT reaction), and then 98 °C for 5 min (inactivation of RT), and then cooled at 4 °C. Polymerase chain reaction (PCR) was performed by a LightCycler^®^ Nano System (Roche Diagnostics K.K., Tokyo, Japan). From the prepared cDNA, a 2.0 µL sample was mixed with sterilized distilled water (7.16 µL), THUNDERBIRD^TM^ SYBR^®^ qPCR Mix (Toyobo) (10 µL), 10 µM sense primer (0.4 µL), 10 µM anti-sense primer (0.4 µL), and 50 × ROX reference dye (Toyobo) (0.04 µL). The initial denaturation period was 95 °C for 1 min, followed by 45 cycles of amplification (95 °C for 10 s, and 60 °C for 30 s). The sequences of the primers are shown in Table 1.

**Table 1:** Sequences of primers used to amplify different genes by real-time PCR.

Target gene (Amplicon size)	Oligonucleotide sequence	Reference
OATP1B1 (248 b.p.)		*
Sense Primer	5′-TGA ATG CCC AAG AGA TGA TG-3′	
Antisense Primer	5′-TGG TGG ACC ACT TTA TAC AC-3′	
OATP1B3 (194 b.p.)		*
Sense Primer	5′-TTG CCG GCC TAA CCT TGA C-3′	
Antisense Primer	5′-TGG TTC CCA CTG ACT TTC ATC AC-3′	
OATP2B1(107 b.p.)		^28^
Sense Primer	5′-TGA TTG GCT ATG GGG CTA TC-3′	
Antisense Primer	5′-CAT ATC CTC AGG GCT GGT GT-3′	
BCRP (162 b.p.)		^29^
Sense Primer	5′-GGA TGA GCC TAC AAC TGG CTT-3′	
Antisense Primer	5′-CTT CCT GAG GCC AAT AAG GTG-3′	
MDR1 (188 b.p.)		*
Sense Primer	5′-GTG GTG GGA ACT TTG GCT G-3′	
Antisense Primer	5′-TAC CTG GTC ATG TCT TCC TCC-3′	
MRP2 (83 b.p.)		^30^
Sense Primer	5′-ACT TGT GAC ATC GGT AGC ATG GA-3′	
Antisense Primer	5′-AAG AGG CAG TTT GTG AGG GAT GA-3′	
β2M (85 b.p.)		*
Sense Primer	5′-TGC TCG CGC TAC TCT CTC TTT C-3′	
Antisense Primer	5′-TTC TCT GCT GGA TGA CGT GAG TAA-3′	

^*^We synthesized these by using Primer Express^®^ software.

The fluorescence of the SYBR green dye was determined as a function of the number of PCR cycles, and the threshold cycle (C_T_), the cycle number at which the amplification reached a significant threshold, was determined. The C_T_ values were used to quantify the PCR products; in other words, the relative expression of the target gene was expressed as 2^−ΔCT^, and ΔC_T_ was calculated by ΔC_T_ = C_T tar_ − C_T β2M_, where C_T tar_ and C_T β2M_ are the C_T_ of the target gene and C_T_ of β2M, respectively.

### Statistical analysis

Measured and IC_50_ values were expressed as the mean ± standard error (S.E.) and median (95% confidence intervals), respectively. Non-overlapping confidence intervals of the IC_50_ were considered statistically significant; and significant differences between two groups were determined by using an unpaired Student’s *t*-test; p values of less than 0.05 was considered statistically significant.

### Ethical approval and informed consent

All procedures performed in studies involving human participants were in accordance with the ethical standards of the institutional and/or national research committee and with the 1964 Helsinki declaration and its later amendments or comparable ethical standards. For this type of study, formal consent is not required.

## Data Availability

All data generated or analyzed during the currently study are available from the corresponding author on reasonable request.
